# Nutritional status of children under 5 years of age in the Brazilian Western Amazon before and after the Interoceanic highway paving: a population-based study

**DOI:** 10.1186/1471-2458-13-1098

**Published:** 2013-11-28

**Authors:** Alanderson A Ramalho, Saulo AS Mantovani, Breno M Delfino, Thasciany M Pereira, Antonio C Martins, Humberto Oliart-Guzmán, Athos M Brãna, Fernando LCC Branco, Rhanderson G Campos, Andréia S Guimarães, Thiago S Araújo, Cristieli SM Oliveira, Cláudia T Codeço, Pascoal T Muniz, Mônica da Silva-Nunes

**Affiliations:** 1 Centro de Ciências da Saúde e do Desporto, Universidade Federal do Acre. Campus Universitário, BR 364, Km 04, Bairro Distrito Industrial, Rio Branco, AC, Brazil; 2 Scientific Computing Program, Avenida Brasil, 4365, Manguinhos, Rio de Janeiro, RJ, Brazil

## Abstract

**Background:**

The aim of this study was to analyse the prevalence of undernutrition, overweight and associated factors, before and after the implementation of the Interoceanic Highway.

**Methods:**

A population-based cross-sectional study on children under 5 years of age was conducted in the municipality of Assis Brasil, AC, Brazil, in 2003 and 2010. Prevalence of undernutrition was observed by using height-for-age Z-scores (HAZ) and adopting a cut-off point equal to or lower than a -2 Z-score. Overweight prevalence was defined by a cut-off point equal to or greater than a +2 Z-score of the WHZ index. Z-scores were calculated relative to WHO 2006 reference data. Semi-structured questionnaires were applied to the children’s guardians, investigating family socio-economic and demographic characteristics, morbidities, access to services and child care. Associated factors were identified by hierarchical multiple logistic regression analysis.

**Results:**

The prevalence of low HAZ (undernutrition) was 7.0% in 2003 and 12.2% in 2010. The prevalence of high WHZ (overweight) was 1.0% and 6.6% for 2003 and 2010, respectively. It was not possible to adjust the multiple model for the year 2003. The factors associated with low HAZ in 2010 were: wealth index, the situation of living with biological parents, maternal height and presence of open sewage, whereas the factors associated with a high WHZ in the same year were: child’s age, mother’s time of residence in the location, mother’s body mass index.

**Conclusions:**

Overweight increase within this undernutrition scenario reveals that the process of nutritional transition began in this Amazonian city only in the last decade, and therefore, it is delayed when compared to overweight in other parts of Brazil. Such nutritional transition in Assis Brasil may have been facilitated by the construction of the Interoceanic Highway.

## Background

The period between weaning and the age of five is nutritionally regarded as the most vulnerable period of the life cycle because that is when rapid growth, loss of passive immunity and the development of the immune system against infection occur [1,2]. Additionally, environmental changes can also affect child nutrition. Thus, monitoring the nutritional status of children is a fundamental instrument for measuring the population’s health [3].

In recent decades, Brazil has recorded a decline in child malnutrition and an increase in overweight [3,4]; however, there are significant regional disparities. In the Northern region of Brazil, where the state of Acre is located, the prevalence of low height-for-age (HAZ) and high weight-for-height (WHZ) have been 14.8% and 6.2%, respectively [5] whereas the national rates for such prevalence are 6.7% low HAZ and 7.3% high WHZ.

An important characteristic that may explain this difference is the geographic isolation that the inhabitants of the Amazon region are exposed to due to the lack of roads. In the state of Acre, there are still several municipalities that have no road access and can be reached only by air or water. To reverse this geographic isolation, several roads have been built in the Amazon region, and in 2001, the Interoceanic Road began to be paved, linking Brazil, Peru and Bolivia, with an estimated cost of US$ 810 million [6].

Until 2000, the state of Acre had only one paved road, and the transit of people and food between some cities could take up to one week. This was the case of the municipality of Assis Brasil, which was subject to geographic isolation. With the construction of the Interoceanic Highway between 2001 and 2003, this situation began to change, and physical access to this city from the capital changed from 7 days to 4 hours.

In January 2003, while there were still twenty kilometres of unpaved road in the Interoceanic Highway in the Brazilian perimeter, we performed a nutritional assessment of the children’s population under 5 years of age in the urban area of Assis Brasil aiming to determine the prevalence of malnutrition in an isolated Amazonian population [7]. Seven years after the completion of paving of this international road, we reassessed the prevalence of malnutrition undernutrition and overweight) in this city in order to evaluate a possible relationship between changes in geographic isolation and factors associated with malnutrition.

## Methods

### Study area

**Figure 1:**
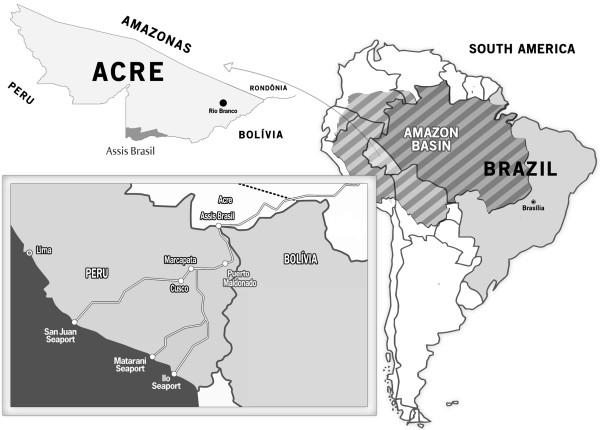
**Map showing the location of Brazil, the Amazon, Acre state and the municipality of Assis Brasil.** The map inside the box shows the Interoceanic Highway connecting Brazil to the Pacific Ocean.

Assis Brasil is located in the Acre River Valley, and it is 344 miles southwest of Rio Branco, the capital of Acre state (Figure 1). It occupies an area of 4,974 km^2^, and it borders the municipality of Brasileia to the east, the cities of Iñapari (Peru) and Bolpebra (Bolivia) to the south, and the municipality of Sena Madureira to the north.

In 2000, Assis Brasil had a total population of 3,264 people (1,708 males and 1,556 females). Of these, 13.39% were aged 0–4 years. For the year 2003, the population was estimated in 3,668 residents. In 2010, the total population increased to 6,017, (3,057 males and 2,960 females), of whom 12.76% were aged 0 to 4 years [8].

### Study design and population

The population investigated consisted of a census with 200 children under 5 years of age living in the urban areas of Assis Brasil, state of Acre, Brazil, in 2003 and 388 children in 2010. These children were located by using the census records of the only Public Health Unit, which included all children at this age living in the urban area. The presence of diseases that would make anthropometric measurement impossible was the exclusion criterion.

Data collection occurred in January and February 2003 and 2010 by means of semi-structured questionnaires and in order to investigate socio-economic characteristics, environmental characteristics, maternal and paternal characteristics, gestational characteristics, child access to services and care, characteristics of childbirth and breastfeeding, self-reported morbidities; diagnosis of anaemia; diagnosis of intestinal parasites.

Both in 2003 and 2010, data about family and the family head’s income were collected. However, this information was unsuitable for analysis due to several reasons (inconsistent income due to temporary jobs, refusal to inform about income or unknown incomes of all family members and incomes in the form of goods instead of money). Therefore, we chose to design a household wealth index, as validated by Filmer and Pritchett for urban areas [9], which is described in the statistical section.

A digital pediatric scale (Soehnle®) with accuracy of 10 g and a maximum capacity of 16 kg was used for the measurement of body weight of children under 2 years. For children over 2 years, we used a portable digital scale (Plenna®) with precision of 100 g and maximum capacity of 150 kg. The length of children under 2 years was obtained by a portable infantometer with accuracy of 0.1 cm placed on a flat surface. The height of children over 2 years was measured by using a wooden stadiometer with accuracy of 0.1 cm fixed to a wall without skirting boards at an angle of 90° to the ground. All anthropometric measurements were performed in duplicate. When the two measurements were discrepant, a third measurement was taken, and the two closest were selected. For analysis, the mean value of duplicates generated indicators in the form of height-for-age (HAZ) and weight-for-height (WHZ) Z-scores. Values from the World Health Organisation [10] were used as a reference and calculated by using WHO Anthro software v.3.2.2 (Department of Nutrition, WHO, Geneva). The cut-off points were: for undernutrition ≤ -2 HAZ and for overweight ≥ +2 WHZ [11]. Extreme values below -6 Z-score and above +6 Z-score were excluded from the analyses. The same routine applied for children over 2 years was used for measuring the mothers’ weight and height; the body mass index value established by the World Health Organisation was used as reference [12]. In 2003, one child was excluded from the research for presenting values below -6 Z-score for the anthropometric indices evaluated, and in 2010, 10 children did not complete anthropometric examination.

Both the interviews and the anthropometric examination were performed by the authors. In 2003, MdaSN and TSA conducted the interviews; a nutritionist (PTM) trained a nurse (TSA) and both performed the anthropometric evaluation. In 2010, new interviewers (medical students listed as authors) trained by MdaSN and TSA conducted the interviews, and two nutritionists (AAR and PTM) and two medical students (trained by AAR, PTM and TSA) performed the anthropometric evaluations.

### Statistical analysis

The socio-economic index was obtained by Principal Component Analysis (PCA), using the XLSTAT software, version 7.5.2 (Addinsoft, New York, NY) and parameters Covariance (n-1) and Correlation biplot/Coefficient = n, as described by Filmer and Pritchett [9]. The household wealth index was created based on the presence of twenty-one consumer goods and household appliances (television set, music system, DVD player, gas stove, refrigerator, washing machine, telephone, bicycle, blender, electric iron, car, sofa, satellite dish, mobile phone, motorcycle, computer, boat, motor boat, water well, power generator and microwave oven) as described in previous publications/studies [7,9,13]. The Jolliffe method adapted for the covariance matrix [14] was used to exclude unimportant variables. For 2003, only five variables remained in the analysis, and for 2010 thirteen variables were maintained in the score. The first principal component explained 61.11% of total variance in 2003 and 30.06% of total variance in 2010. The scores for each variable were added to estimate the household wealth index, which was stratified in two groups (the poorer half and the richer half). For each year, this procedure was performed for all children versus only one child per household in order to check for non-independence of individual information. The comparison of the indices calculated by each technique showed no statistical differences, thus we opted to use the index per household instead of the index per child.

*Exploratory analysis.* A database was created with SPSS 13.0 software (SPSS Inc., Chicago, IL). The distribution of the independent variables was identified by using the *Kolmogorov*-Smirnov test. Student’s T test was used to compare means, and the chi-square test was utilized to compare frequencies or proportions with α = 0.05 as the critical level for years 2003 and 2010. Exploratory univariate logistic regression analysis, using the R software version 2.14.0 (The R Foundation for Statistical Computing), examined potential risk factors and confounders, including categorical individual-level variables, continuous individual level variables, categorical household-level variables and continuous household-level variables for 2003 and 2010.

The factors associated with undernutrition and high overweight were identified by hierarchical multiple logistic regression analysis using the conceptual model (Table 1) and adapted procedures from previous studies [15-17]. Separate multiple logistic regression models for the year 2010 were fitted to individual- and household-level variables using manual inclusion and following from distal to proximal blocks of variables. Covariates were maintained in subsequent multivariate models, together with the *a-priori* potential confounders (age and gender), if they were associated with the outcome, in exploratory unadjusted analysis, at a level of significance of 20%, in each block. It was not possible to adjust the multiple model for 2003, and for this year, we presented the results of the univariate analyses only.

**Table 1:** Conceptual model for hierarchical analysis of factors associated with overweight and malnutrition

**Distal**	**Block 1: Environmental and socio-economic**
	*Socio-economic variables*
	Wealth index
	Household type
	House floor type
	Home ownership
	Receipt of benefits
	Food production for own consumption
	*Environmental Variables*
	Waste collection
	Presence of open sewage
	Presence of electric power supply
	Water well or drinking water treatment Toilet type
**Intermediate**	**Block 2: Maternal and paternal characteristics**
	Age
	Gender
	Marital status
	Skin colour
	Schooling
	Paid work
	Smoking
	Consumption of alcoholic beverages
	Time living in the municipality
	Maternal height
	Maternal body mass index
	**Block 3: Gestational characteristics**
	Number of pregnancies
	Pre-natal care
	Morbidity during pregnancy
	**Block 4: Access to services and child care**
	Living with biological parents
	Attending nursery or school
	Monitoring through health care service
**Proximal**	**Block 5: Characteristics of child birth and breastfeeding**
	Birth weight
	Duration of exclusive breastfeeding
	Duration of total breastfeeding
	**Block 6: Morbidity**
	Self-reported morbidities
	Hospitalisation at least once in life
	Diagnosis of anaemia
	Diagnosis of intestinal parasites

Adapted from UNICEF [20].

The goodness of fit was assessed by analysis of variance, Akaike’s information criterion (AIC) values or odds ratio (OR) changes. The existence of significant interaction terms was evaluated and none was found. Diagnostic tests were applied to identify outliers, using the R 2.14.0 software package. No outliers (important influential points) were identified for any of the variables included in the final model.

Additional analyses were performed using the exact logistic regression package *erlm* in order to approximate exact conditional inference for logistic regression models presenting a small number of events (less than 30 events) [18]. We also used mixed the effects of logistic regression (MASS library of R program) to explore the association between individual and household-level covariates and the occurrence of undernutrition and overweight, taking into account the nested structure of the data (some of the children shared the same household). Results were very similar to those obtained with the previous generalised linear model with fixed effects only. Both models resulted in similar OR estimates and similar confidence intervals to those shown by the logistic regression model described earlier, and therefore, results are not presented.

### Ethical considerations

The study was approved by the Ethics Committee for Experimentation with Human Beings at the University of São Paulo (810/2002) and the Federal University of Acre (23107.014335/2009-69). Informed consent was obtained from each participant’s legal guardian.

## Results

### Comparison of the city before and after the construction of the Interoceanic Road

Between 2003 and 2010, some important economic, social and demographic changes occurred in the city. There was an increase in the number of elementary schools and teachers, and a new university campus was opened. For the first time, the city received offices of several public and private agencies, such *Banco do Brasil*, the Federal Police, the Federal Revenue Agency, the National Health Surveillance Agency, the Reference Center for Social Security and a Prosecutor’s Office. A new Basic Health Unit for the rural area was implemented in 2006. Between 2005 and 2010 new health care services started to be offered for free, such as screening for metabolic diseases and monitoring of hypertension and diabetes, besides the programs already in place in the county since 2003.

### Parental and children’s socio-economic characteristics in 2003 and 2010

Table 2 shows the distribution of children studied by year of the population census, according to family socio-demographic and economic characteristics. The population distribution by gender and age were similar in both periods. Females accounted for 47% of children in 2003 and 50.8% in 2010 (p = 0.386). The average age was 2.43 years in 2003 and 2.43 years in 2010 (SD 2003: 1.37, SD 2010: 1.47, t-test: 0.997).

**Table 2:** Distribution of children (%) under 5 years according to socio-demographic and economic characteristics

	**2003**		**2010**		
	**(n = 200)**		**(n = 388)**		
**Variables**	N*	**%**	N*	**%**	p-value**
***Gender***					
Male	94	47.0%	197	50.8%	0.386
Female	106	53.0%	191	49.2%	
***Age***					
Under 1 year	35	17.5%	81	20.9%	0.499
From 1 to 2 years	45	22.5%	86	22.2%	
From 2 to 3 years	42	21.0%	60	15.5%	
From 3 to 4 years	43	21.5%	86	22.2%	
From 4 to 5 years	35	17.5%	75	19.3%	
***Predominant material in the house floor***					
Cement, brick, ceramic tile, slab-stone	32	16.0%	112	28.9%	**0.001**
Wood or soil	168	84.0%	275	71.1%	
***Electric power supply at home***					
Lacking	22	11.0%	12	3.1%	**< 0.001**
Present	178	89.0%	376	96.9%	
***Drinking water treatment***					
Without treatment; lack of mineral water	65	32.5%	135	34.8%	**< 0.001**
Mineral water without treatment	15	7.5%	131	33.8%	
Boiled, strained or filtered	84	42.0%	84	21.6%	
Chlorinated	36	18.0%	38	9.8%	
***Waste disposal***					
Council collection	179	89.5%	370	95.4%	**0.024**
Buried or burned	8	4.0%	8	2.1%	
Dumped in land or river	13	6.5%	10	2.6%	
***Open sewage***					
Lacking	161	80.9%	235	60.6%	**< 0.001**
Present	38	19.1%	153	39.4%	
***Sanitary installation***					
Toilet (with running water)	31	15.5%	242	62.4%	**< 0.001**
Pit toilet	128	64.0%	110	28.4%	
Lacking sanitary installations	41	20.5%	36	9.3%	
***Production of food for own consumption***					
No	84	71.8%	177	45.6%	**< 0.001**
Yes	33	28.2%	211	54.4%	

Assis Brasil, AC, 2003 and 2010.

*Missing in some variables **Person’s chi-square test.

By comparing the periods, it is observed that there was an increase in the use of cement, bricks, ceramic tiles or stone-slab as the predominant materials for house floor finishing (16% to 28.9%, p <0.001); the presence of electric power supply at home (89% to 96.9%, p <0.001); the acquisition of mineral water (7.5% to 33.8%, p <0.001) and consequent decrease in drinking water treatment. An increase in open sewage systems (19.1% to 39.4%, p <0.001), the number of sanitary facilities with running water (15.5% to 62.4%, p <0.001), food production for household consumption (28.2% to 54.4%, p <0.001) and household waste collection by the public system (89.5% to 95.4%, p = 0.024) was also observed. No difference household type was observed (p = 0.138).

Table 3 illustrates the distribution of children younger than 5 years, according to parental characteristics in the years 2003 and 2010. By comparing these periods, a rise in parental schooling above 8 years of school attendance (father: 42.9% to 66.2%, p <0.001 and mother: 40.2% to 49.6%, p <0.001), father’s paid work in the last 30 days (91.1% to 63.1%, p <0.001), maternal overweight (26.9% to 47.3%, p <0.001) and mother’s average age (26 to 28 years, p = 0.005) was observed. There was no statistically significant difference for years 2003 and 2010 as regards average maternal height (about 159 cm, p = 0.916), maternal marital status (p = 0.627) or mother’s paid work in the last 30 days (p = 0.094). In 2010, the median length of maternal residency in the community was 15 years (minimum 2 weeks, maximum78 years), and 58.5% of the mothers had been living in the city for longer than 7 years. This information was not obtained for the year 2003.

**Table 3:** Distribution of children (%) under 5 years according to paternal characteristics

	**2003**		**2010**		
	**(n = 200)**		**(n = 388)**		
**Categorical variables**	N*	**%**	N*	**%**	p-value**
**Continuous variables**^**#**^		**Mean(SD)**		**Mean(SD)**	
***Father’s schooling (years)***					
Did not go to school	15	8.2%	-	-	**< 0.001 '**
1 to 4 years inclusive	50	27.5%	70	20.1%	
5 to 8 years inclusive	39	21.4%	48	13.8%	
More than 8 years	78	42.9%	231	66.2%	
***Father’s paid work in the last 30 days***					
No	17	8.9%	81	20.9%	**< 0.001**
Yes	173	91.1%	245	63.1%	
***Maternal marital situation***					
Lack of a partner	26	22.2%	93	24.4%	0.627
Presence of a partner	91	77.8%	288	75.6%	
***Maternal schooling(years)***					
Did not go to school	8	6.8%	-	-	**< 0.001 '**
1 to 4 years inclusive	27	23.1%	88	25.2%	
5 to 8 years inclusive	35	29.9%	88	25.2%	
More than 8 years	47	40.2%	173	49.6%	
***Mother’s paid work in the last 30 days***					
No	67	59.3%	259	67.8%	0.094
Yes	46	40.7%	123	32.2%	
***Maternal age***^**#**^					
Average (years)	183	26.21 (7.64)	378	28.43 (9.33)	**0.005**
***Maternal height***^**#**^					
Average (cm)	187	158.5 (5.29)	350	158.8 (39.63)	0.916
***Maternal BMI***					
Below 25 kg/m^2^	128	73.1%	184	52.7%	**< 0.001**
Equal or above 25 kg/m^2^	47	26.9%	165	47.3%	
***Maternal time of residence in the urban area***					
Less than 1 year	-		43	11.1%	**-**
Between 1 and 7 years			107	27.6%	
7 years or more			227	58.5%	

Assis Brasil, AC, 2003 and 2010.

*Missing in some variables.

**p-values without a mark ( ): Pearson’s chi-square test; with a mark ( ' ): Fisher's exact test.

^#^Continuous variables.

Table 4 shows the characteristics of children under 5 years of age in the two studied years, according to individual socio-demographic, birth and breastfeeding characteristics, and morbidities. When comparing results for 2003 and 2010, there was no significant difference in delivery type (p = 0.065), low birth weight (p = 0.085), breastfeeding at birth (p = 0.158), duration of exclusive breastfeeding (p = 0.123) and the situation of living with biological parents (p = 0.501). However a reduction in the number of diarrhoea cases in the last 15 days (34.5% to 18.2%, p <0.001) and an increased number of children hospitalized at least once in life were observed (19.3% for 37.2%, p <0.001).

**Table 4:** Distribution of children (%) under 5 years according to socio-demographic, birth and breastfeeding characteristics, and morbidities

	**2003**		**2010**		
	**(n = 200)**		**(n = 388)**		
**Categorical variables**	N*	**%**	N*	**%**	p-value**
***Living with biological parents***					
Living with biological mother and father	139	69.5%	259	66.8%	0.501
Living with only one biological parent or neither one	61	30.5%	129	33.2%	
***Delivery type***					
Natural	146	86.4%	292	79.8%	0.065
C-section	23	13.6%	74	20.2%	
***Birth weight***					
Above 2,500 g	166	94.9%	305	90.5%	0.085
Below or equal to 2,500 g	9	5.1%	32	9.5%	
***Breastfeeding at birth***					
Lacking	11	5.6%	12	3.2%	0.158
Present	184	94.4%	364	96.8%	
***Duration of exclusive breastfeeding †***					
More than 30 days	114	64.0%	159	56.8%	0.123
Equal to or less than 30 days	64	36.0%	121	43.2%	
***Diarrhoea in the last 15 days***					
Lacking	131	65.5%	315	81.8%	**< 0.001**
Present	69	34.5%	70	18.2%	
***Hospitalisation at least once in life***					
No	159	80.7%	243	62.8%	**< 0.001**
Yes	38	19.3%	144	37.2%	
***Anaemia diagnosis***					
No	69	49.6%	252	72.2%	**< 0.001**
Yes	70	50.4%	97	27.8%	
***Parasitosis diagnosis***					
No	110	68.3%	207	62.3%	0.194
Yes	51	31.7%	125	37.7%	
***Has lived in rural or riverine area before***					
No	197	98.5%	363	93.6%	**0.007′**
Yes	3	1.5%	25	6.4%	
***Child length of time living in the urban area***					
Since birth	181	90.5%	311	80.2%	**0.001**
After birth	19	9.5%	77	19.8%	

Assis Brasil, AC, 2003 and 2010.

*Missing in some variables.

**p-values without a mark ( ): Pearson’s chi-square test; with a mark ( ' ): Fisher's exact test.

^†^Excluding children that were only breastfed.

The percentage of children that had lived in rural or riverine areas prior to moving to the urban area increased (1.50% to 6.40%, p = 0.007). As this may suggest a migration movement from rural and riverine areas to the urban area of Assis Brasil, the study population was further stratified according to place of residence at birth (urban or not urban). For the year 2003, there were no significant differences. In 2010, children that had lived outside the urban area prior to the study showed some worsened socio-economic features, such as more houses with a wooden floor (p = 0.024), lack of electric power in the house (p = 0.001); suboptimal drinking water treatment (p = 0.006) and lower maternal schooling (p = 0.043).

### Prevalence of anthropometric extremes

Table 5 shows the prevalence of anthropometric indicators of undernutrition and overweight for children under 5 years, according to the studied years. By comparing the two periods, significant differences in high WHZ were observed. The prevalence of high WHZ increased (1.0% to 6.6%, p = 0.002), but there were there was no difference in the prevalence of low HAZ (7% to 12.2%, p = 0.055).

**Table 5:** Prevalence (%) of anthropometric indicators of children under 5 years

	**2003**		**2010**		
	**(n = 199)**		**(n = 378)**		
**Indicators**	**n**	**%**	**n**	**%**	p-value*
***Low height-for-age (stunting)***					
No	185	93.0%	332	87.8%	**0.055**
Yes	14	7.0%	46	12.2%	
***High weight-for-height (overweight)***					
No	197	99.0%	353	93.4%	**0.002'**
Yes	2	1.0%	25	6.6%	

Assis Brasil, AC, 2003 and 2010.

*p-values without a mark ( ): Pearson’s chi-square test; with a mark ( ' ): Fisher's exact test.

Due to socio-economic differences according to the time when the children had been living in the municipality, the prevalence of anthropometric indicators was also stratified by this variable. For the year 2003 no significant differences were identified. In 2010, about 23.3% of the children who had not been living in the urban area since birth showed a height-for-age deficit (p = 0.002).

### Undernutrition

Table 6 shows the prevalence and factors associated with undernutrition in 2003 by univariate logistic regression. Table 7 shows the factors associated with undernutrition in the studied population in 2010 obtained by hierarchical multiple analysis. From the first block, variables wealth index (poorer half, OR: 5.87; CI 95%: 2.33 to 14.76) and open sewage (OR: 2.27; CI 95%: 1.08 to 4.72) remained in the model. From the second block, only maternal height (lower half, OR: 3.53, CI 95%: 1.58 to 7.86) remained associated with undernutrition. In the fourth block, only variable living with biological parents remained in the model (living with only one parent or none, OR: 2.27, CI 95%: 1.08 to 4.76). No variables from the third, fifth and sixth blocks remained in the model. No concomitant morbidities remained in the final model. The gender and age variables were used in the model as controls. Results from a separate model stratified by place of birth (urban x rural) showed similar results to those in the main model, suggesting that although the prevalence of undernutrition in children that had lived part of their lives outside the urban area of the city was high, all other factors associated with undernutrition remained the same.

**Table 6:** Prevalence (%) and Odds Ratio (OR) of low height-for-age in children under 5 years according to socio-demographic and economic characteristics, 2003

**Categorical variables**	N* (n = 199)	**%**	**Crude**	p value**
**Continuous variables**^**#**^		**Mean(SD)**	**OR**	
***Home ownership***				
Own home (fully owned or mortgaged)	162	4.9%	1	0.027
Rented, gifted or invaded	37	16.2%	3.726	
***Electric power supply***				
Lacking	22	22.7%	1	0.011 '
Present	177	5.1%	0.182	
***Wealth index***				
Richer half	104	1.9%	1	0.004 '
Poorer half	95	12.6%	7.373	
***Mother schooling (years)***				
Did not go to school	8	37.5%	1	< 0.001
1 to 4 years	27	7.4%	0.013	
Above 4 years	81	1.2%	0.002	
*p linear tendency*				*0.003*
***Maternal height (cm)***^***#***^	186	158.5 (5.29)	0.855	0.01
***Number of pregnancies***^***#***^	113	2.63 (2.21)	1.35	0.018
***Anaemia diagnosis***				
No	69	49.64%	1	0.176
Yes	70	50.36%	3.09	

Assis Brasil, AC, 2003.

*Missing in some variables.

**p-values without a mark ( ): Pearson’s chi-square test; with a mark ( ' ): Fisher's exact test.

**Table 7:** Factors associated with a low height-for-age in children under 5 years obtained by hierarchical multiple analysis, 2010

**Variables**	**Crude OR**	p value**	Adjusted OR*	**(CI 95%)**	p value**
***Wealth index***					
Richer half	1		1		
Poorer half	7.2	< 0.001	5.87	(2.33 – 14.76)	< 0.001
***Open sewage***					
Lacking	1		1		
Present	2.82	0.001	2.27	(1.08 – 4.72)	0.029
***Living with biological parents***					
Living with biological father and mother	1		1		
Living with one biological parent or neither one	2.56	0.004	2.27	(1.08 – 4.76)	0.031
***Maternal height (cm)***					
Taller half (156.5 to 178.5)	1		1		
Shorter half (138.8 to 156.5)	3.72	< 0.001	3.53	(1.58 – 7.86)	0.002
***Anaemia diagnosis***					
No	1	0.003	-	-	-
Yes	2.74				
***Parasitosis diagnosis***					
No	1	0.54	-	-	-
Yes	1.25				

Assis Brasil, AC, 2010.

*Odds Ratio (OR) adjusted by all variables in the table, gender and age.

**Wald test.

### Overweight

Factors associated with overweight in children under 5 years, obtained through hierarchical multiple regression analysis are presented in Table 8. From the second block variables, maternal BMI (≥ 25 kg/m^2^, OR: 3.31, CI 95%: 1.25 - 8.75) and the mother’s time of residency in the municipality (years of residence, OR: 1.07, CI 95%: 1.03 to 1.11) remained associated with overweight. Among the variables of the first, third, fourth, fifth and sixth blocks, none remained in the model. Variables gender and age (in years, OR = 0.58, CI 95%: 0.38 to 0.87) were added to the model as controls.

**Table 8:** Factors associated with high weight-for-height in children under 5 years obtained by hierarchical multiple analysis, 2010

**Variables**	**Crude OR**	p value**	Adjusted OR*	**(CI 95%)**	P value**
***Child age***					
Age in years	0.67	0.025	0.58	(0.38 – 0.87)	0.009
***Maternal IBM***					
Below 25 kg/m^2^	1		1		
Equal or above 25 kg/m^2^	2.68	0.034	3.31	(1.25 – 8.75)	0.016
***Mother’s time residing in town***					
Years	1.05	0.002	1.07	(1.03 – 1.11)	< 0.001

Assis Brasil, AC, 2010.

*Odds Ratio (OR) adjusted by all variables in the table and gender.

**Wald test.

## Discussion

Improvements in socio-economic conditions, parental education, water supply, and decrease in the global burden of disease in Assis Brasil were observed between the studied periods. However, population growth in the period from 2003 to 2010 was higher than usual. The municipality did not have adequate urban infrastructure to receive this demand, resulting in increased areas of illegal settlements. This may partly explain the increased presence of open sewage observed when comparing periods.

In this study, undernutrition rates did not change significantly over the decade. In 2003, undernutrition prevalence in Assis Brasil (7.0%) was similar to the Brazilian average of 8.7% [19], but while in 2006 the national average decreased to 6.7% [5], in the city of Assis Brasil, undernutrition rates remained high (12.2%) until 2010.

Obesity occurs more often in the first years of the life cycle, between 5 and 6 years of age, and in adolescence [20,21]. In the present study, the prevalence of overweight increased from 1% to 6% between 2003 and 2010, reaching the national average identified in PNDS 2006 [5]. This may reflect an increasing growth in children’s overweight and a future public health problem for the region. A possible explanation for this phenomenon is undernutrition early in life due to poor maternal nutrition and inadequate nutrition in early childhood, predisposing the body to store fat when eating foods rich in carbohydrates and fats. [22].

Another explanation for the overweight increase is the nutrition transition. The name “ nutrition transition” means a change in nutritional patterns related to food intake resulting from changes in social, economic and demographic status that affect health [23]. According to Batista Filho and Rissin [24], the nutrition transition is a process characterised by four stages. In the first stage, the disappearance of acute and severe protein malnutrition related to protein intake deficiency is observed. In the second stage the disappearance of protein-energy malnutrition characterised as insufficient intake of calories and nutrients occurs. The third phase is characterised by the appearance of the binomial overweight/obesity on a population scale, caused by excessive food intake associated with increasing sedentariness. And the last stage of the transition is set in the correction of short stature, since there is no longer a poor intake of nutrients [24].

Several authors have reported that the process of demographic and economic transition observed in developing countries such as Brazil, contributes to the nutrition transition [23-25]. Reported studies show that Brazil is currently in the fourth stage of the nutritional transition process. However, this process is not uniform, and Assis Brasil, which is located in an underdeveloped region of the country, is still between the second and third stages, with increased overweight rates, but sustained chronic undernutrition. This is suggested by the high prevalence of low HAZ and high WHZ, as found in this study.

The process of nutritional transition is marked by the intake of a high calorie diet, rich in saturated fat and refined carbohydrates, which are characteristics of most industrialized foods, as well as by low intake of complex carbohydrates and fibers. The implementation of the Interoceanic Highway may have promoted the arrival of new foods from other regions of Brazil and Peru. When assessing the perception of the changes brought by BR-137 (Transoceanic Highway) to Assis Brasil residents in 2010, Martins et al. (unpublished observations) found that 89.7% of respondents reported improvements in the variety and availability of food and 8.8.5% reported improvements in the local market. Poor logistic access to Assis Brasil before the BR-317 was related to the unavailability of perishable foods at home. In addition, preliminary results from the Feeding Infants and Toddlers Study (FITS) confirm an excessive intake of industrialised food by children at this age [26].

Factors associated with undernutrition were mostly related to socio-economic status (wealth index, living conditions and schooling and number of pregnancies) and maternal height. Similar findings from 47 countries suggested that low height-for-age led to a greater association with socio-economic inequality than did low weight-for-height [27]. A comparison of national nutritional surveys corroborate this information, describing that 21.7% of the reduction in the prevalence of child malnutrition between 1996 and 2006 can be attributed to the increasing purchasing power of Brazilian families. The government is partly responsible for this increase by granting benefits to mothers or guardians in poorer families [3].

The presence of open sewage was associated at a distal level with undernutrition in 2010. This has been reported elsewhere [28,29]. Souza et al. (2012), while assessing malnutrition in two municipalities in the state of Acre in 2003, found that children exposed to open sewage near home were more likely to show low height-for-age in relation to the unexposed ones [17]. When analysing national surveys, Monteiro et al. [30] also confirmed the association between inadequate sanitation and undernutrition. The possible biological relationship between undernutrition and open sewage is the increase in the number of cases of diarrhoea and intestinal parasitosis as well as other morbidities, resulting in growth retardation.

For the year 2003, poor maternal education levels showed greater association with low height-for-age than did socio-economic variables. Other studies on the relation between social and environmental conditions and malnutrition in São Paulo, Belo Horizonte, Maceió and Rio Grande do Sul also showed this association [31-34]. This relationship may result from the basic information on the importance of personal and household hygiene habits and practices of adequate nutrition for child growth and development. Drachler et al. [35] reported that the mother figure represents the bond between children and the environment, besides the fact that it is also the mother who usually decides on her family’s eating habits and on hygiene and immunization care. In 2010, an increase in maternal educational levels was observed, and it was not associated with undernutrition anymore.

In this study, for the year 2003, each new pregnancy increased the chance of an already existing child under 5 years of age to develop low height-for-age by 35%. Eastwood and Lipton [36] showed that, in families with low purchasing power, the impact of high fertility on the family income was more pronounced. Therefore, multiple pregnancies may have had an impact on socio-economic conditions in 2003, thus contributing to less food available and higher levels of undernutrition.

The effect of maternal height in child height can be explained as a biological relationship and, at the same time, as the result of socio-economic long-standing unfavorable conditions that had affected the mother in the past and are presently affecting their children as well. Previous studies have also shown this association between maternal height and stunting [31,37,38], and between maternal height and poverty and adverse socio-environmental conditions. Therefore, maternal short stature can predict undernutrition in children [31,37,38].

Although the prevalence of anemia and intestinal parasitosis was higher in undernourished children, these two morbidities were not associated with undernutrition in the final model possibly because they have similar associated factors and are not the cause of undernutrition per se. Some studies have already reported that inadequate sanitary conditions are associated with anaemia [39,40] as well as undernutrition in children [28,29].

Overweight in Assis Brasil was associated with child age, time of residence in the town and maternal BMI. The inverse association between age and overweight, found in this study, has also been reported by other Brazilian studies [41-43]. A study conducted in southern Brazil [42] reported a negative trend between age and overweight and obesity, which is possibly explained by the result of increased physical activity throughout the years, but no confirmatory studies have been published so far.

While low maternal height was associated with undernutrition, maternal overweight was a predictor of child overweight in this study as well. This is a common association reported extensively elsewhere [35,44-49]. According to Maffeis et al. [50], the main risk factor for childhood obesity is still parental' obesity, occurring as a result of genetic representation concomitant with environmental influences. However, the mother is the main agent in determining the dietary habits adopted by a child, since she is the main individual involved in the selection and preparation of food [49].

In 2010, the length of time during which the mother had been living in the municipality was associated with overweight children. Each year of maternal residence in the municipality increased the chance of a child′s being overweight by 7%. The most likely hypothesis would be the rural exodus promoted by the Transoceanic Highway or the migration of people from other municipalities with poor conditions in search of better living conditions and the contact with industrialised food rich in fat and carbohydrates. This hypothesis is supported by three main findings: approximately 19.8% of the children were born outside the urban area and migrated to it when they were between 1 and 5 years old; undernutrition among children born in rural areas was high in this study, and overweight prevalence was higher in children whose families had been living in the urban area for a longer periods.

The main limitation of this study is that the cross-sectional design precludes a cause-and-effect relationship between the studied variables. Therefore, the associated factors observed should be interpreted as associations between events, and not as risk factors. Another limitation is that the population size is small for detecting events and therefore there may be an overestimation of the strengths of the associations described. This must be taken into account when interpreting results. Finally, not all variables studied in 2010 were investigated in 2003, so we may not have been able to detect all possible associated factors in 2003, such as the number of years of maternal residency in the urban area.

## Conclusions

Comparing the years 2003 and 2010, an increase in the prevalence of overweight (high WHA) and in the prevalence of undernutrition (low HAZ) was observed. Factors associated with undernutrition in the municipality are especially related to socio-economic status and maternal characteristics. On the other hand, the progress of overweight in this conservation of undernutrition reveals that the process of nutritional transition began in this Amazonian city only in the last decade, and therefore, it is delayed when compared to nutritional transition in other parts of Brazil. Such transition in Assis Brasil may have been facilitated by the construction of the Interoceanic Highway. Factors associated with overweight in Assis Brasil for the year 2010 were children′s age, mother’s time of residency in the municipality and maternal BMI.

## Competing interests

The authors declare that they have no competing interests.

## Authors’ contributions

M da SN, PTM, AAR, SASM, BMD, TMP, ACM, HOG, AMB, FLCCB, RGC, ASG, TSA and CSMO designed the study and collected the data. All authors analysed the data under the supervision of M da SN, CTC, and PTM. AAR and M da SN wrote the manuscript. All other authors revised the manuscript and contributed to the discussion of the results and revision of the intellectual contents in the research. All authors approved the final version of the manuscript.

## Pre-publication history

The pre-publication history for this paper can be accessed here:

http://www.biomedcentral.com/1471-2458/13/1098/prepub
